# The gut microbiome as a modifiable contributor to autism spectrum disorder: a precision gut–immune–brain perspective

**DOI:** 10.3389/frcha.2026.1900538

**Published:** 2026-08-19

**Authors:** Eva Sclabassi, Jun Hur, Jiying Ling, Yuen Gao

**Affiliations:** 1 Department of Radiology, Michigan State University, East Lansing, MI, United States; 2 College of Nursing, Michigan State University, East Lansing, MI, United States

**Keywords:** autism spectrum disorder, gut microbiome, gut–brain axis, microbiota, probiotics

## Introduction

Autism spectrum disorder (ASD) is a heterogeneous neurodevelopmental condition characterized by differences in social communication, restricted and repetitive behaviors, sensory processing, and developmental trajectories. Although ASD has a strong genetic and developmental basis, genetics alone does not fully explain the wide variation in symptom severity, gastrointestinal symptoms, immune findings, and response to interventions across individuals. Because many children with ASD also experience gastrointestinal symptoms such as constipation, diarrhea, abdominal pain, reflux, and bloating, recent research has increasingly focused on microbiome-related gut–brain pathways as possible contributors to ASD-related biology (1–4). However, current evidence does not support framing the gut microbiome as a single cause of ASD. A more cautious and biologically plausible interpretation is that the microbiome represents a modifiable biological system that may interact with genetic susceptibility, environmental exposures, diet, early development, epigenetic regulation, microbial metabolites, and immune signaling.

In this Opinion article, we argue that ASD microbiome research may benefit from moving away from broad claims that “gut dysbiosis causes autism” and toward a precision gut–immune–brain framework. Recent human studies with larger sample sizes suggest recurring microbial, metabolic, dietary, and biomarker patterns in ASD, while mechanistic animal studies point to possible pathways involving microbial metabolites, CD4+ T cells, microglia, and neuroinflammation (1, 2, 5, 6). Collectively, these findings are consistent with the idea that microbiome monitoring and targeted microbial interventions may eventually be more useful for biologically defined ASD subgroups than for ASD as a single uniform condition.

## Narrative literature approach

Because this article is an Opinion article rather than a systematic review, the literature was selected using a narrative approach. We prioritized recent peer-reviewed studies directly relevant to ASD microbiome research, especially human-centered studies with larger cohorts when available, studies examining microbial function or metabolites, and mechanistic work relevant to gut–immune–brain pathways. Selected intervention studies and reviews were also included to evaluate translational potential and limitations. This approach was intended to synthesize emerging themes rather than provide an exhaustive or quantitatively pooled review.

## Human microbiome findings and the challenge of causation

Recent high-impact human studies have strengthened the evidence that ASD is associated with altered gut microbial communities and microbial function. Su et al. performed metagenomic sequencing in 1,627 children aged 1–13 years and identified altered archaea, bacteria, fungi, viruses, microbial genes, and metabolic pathways in children with ASD (1). Their multikingdom and functional marker panel achieved strong diagnostic accuracy, suggesting that microbiome-based signatures may eventually contribute to noninvasive biomarker development (1). Similarly, Chen et al. integrated gut metagenomic and metabolomic profiles from more than 1,100 children and identified microbial genomic variants and metabolic shifts associated with ASD, further supporting the importance of microbial function in addition to microbial composition (5). Liu et al. extended this concept by showing that bacterial genomic structural variations in 452 children were associated with ASD and could improve diagnostic modeling beyond bacterial abundance alone (6). Together, these studies suggest that ASD-related microbiome differences are not limited to which microbes are present but may also involve what microbial communities are capable of doing biologically.

Most human ASD microbiome studies remain associative. Association indicates that microbial features differ between groups or co-occur with ASD-related traits, but it does not determine whether the microbiome contributes to those traits or reflects diet, gastrointestinal symptoms, medications, or other factors. Mediation would require evidence that microbiome-related changes help explain the relationship between upstream factors, such as diet or immune activation, and downstream behavioral or biological outcomes. Causation requires stronger longitudinal or interventional evidence, which remains limited in humans.

Interpretation is further complicated by heterogeneity in ASD cohorts and microbiome methods. Studies vary in diagnostic criteria, ASD severity, cognitive and language profiles, gastrointestinal symptoms, age, sex, medication exposure, and behavioral phenotype. They also differ in stool collection, sequencing platform, bioinformatic pipeline, taxonomic resolution, metabolomic assessment, and statistical adjustment for confounders. Therefore, microbiome findings in ASD should be interpreted as patterns observed within specific cohorts rather than as a universal microbiome signature (2, 3, 7). Future studies should clearly report how ASD diagnosis was confirmed, such as DSM-based criteria or standardized diagnostic instruments, and should characterize ASD severity, language/cognitive profile, gastrointestinal symptom burden, and medication exposure.

These limitations are especially important because ASD is highly heterogeneous, and the microbiome is strongly shaped by diet, family environment, medication use, geography, and gastrointestinal physiology (2). Morton et al. addressed this complexity through a multi-level analysis across microbiome, diet, metabolomic, cytokine, and brain gene-expression datasets, showing that ASD-associated microbial profiles align with broader biological and dietary patterns (2). Notably, sibling-matched cohorts did not show the same architecture as age- and sex-matched cohorts (2). This means microbiome differences may be biologically meaningful, but they are also deeply embedded in environmental and family contexts.

## Diet, family context, and microbiome interpretation

An important future direction is to study diet not only as a confounder, but also as an active component of the gut–brain framework. Children with ASD often have feeding problems and food selectivity, which may influence microbiome composition and metabolic output. Wu et al. studied 818 children and found ASD-specific diet–microbiome associations, linking poor dietary quality with core autistic symptoms, gastrointestinal complications, atypical eating behaviors, and disrupted microbial connectivity (7). Their study also highlighted synthetic food additives, including polysorbate-80 and carrageenan, as factors associated with altered microbiome networks in ASD (7). These findings are consistent with a more integrated model in which restricted eating, gastrointestinal symptoms, and microbiome differences may reinforce one another.

Family-based studies help distinguish ASD-associated microbial patterns from those shaped by shared genetics and household environment. Lu et al. examined gut microbiome composition and strain-sharing in multiplex ASD families, emphasizing the importance of familial and environmental influences in ASD-related microbial profiles (8). Chen et al. also used a family-member design, comparing children with ASD, non-ASD siblings, and parents, and found differences in microbial diversity and composition while attempting to reduce bias from shared genetic and environmental factors (9). These studies suggest that microbiomes may contribute to ASD-related traits, but they should be studied within the real-life context of diet, household microbial sharing, genetics, and family environment.

Additional early-life and environmental factors should also be considered when interpreting ASD microbiome findings. Antibiotic exposure, proton pump inhibitor use, delivery mode, breastfeeding history, early-life infections, socioeconomic status, geography, and household environment can influence microbiome composition. These variables may interact with food selectivity, gastrointestinal symptoms, and medication use in children with ASD. More complete reporting of these factors would help distinguish ASD-associated microbial patterns from microbiome differences related to lifestyle, medical history, or environment.

## Microbial metabolites as a plausible gut–brain link

Microbial metabolites provide a plausible biological link between gut dysbiosis and ASD-related brain or behavioral traits. Rather than focusing only on which bacteria are increased or decreased, the field may benefit from examining what those microbes are producing. Wang et al. identified an elevated gut microbial GABA/glutamate ratio in preschool children with mild ASD and linked this metabolic imbalance to overrepresented *Escherichia* (10). Their mouse experiments further suggest that *E. coli* overgrowth may potentiate social deficits, connecting human metabolic signatures with mechanistic testing (10). Aziz-Zadeh et al. provided another human mechanistic link by combining fecal metabolomics, fMRI, and behavioral assessments in 43 children with ASD and 41 neurotypical controls (11). They found that tryptophan-related metabolites, including kynurenate, were lower in ASD and associated with altered insular and cingulate activity, ASD severity, disgust propensity, and sensory sensitivity (11).

These findings support a metabolite-centered model of the gut–brain axis. Microbial products such as GABA-related metabolites, glutamate, tryptophan derivatives, short-chain fatty acids, and other neuroactive compounds may influence the neurodevelopmental and behavioral features through circulation, vagal signaling, immune pathways, or barrier function (10, 11). This does not mean that microbial metabolites independently “cause” ASD. Instead, they may help explain why certain children experience stronger gastrointestinal, sensory, immune, or behavioral features, and may offer biologically meaningful targets for subgroup-specific monitoring or intervention.

## Mechanistic evidence from animal models

Although human studies are essential, mechanistic animal studies remain valuable because they allow controlled testing of causality. Park et al. developed a germ-free BTBR mouse model and found that the absence of gut microbiota in male mice reduced several ASD-associated behaviors and decreased inflammatory brain-resident T cell populations (12). They also showed that CD4+ T cell depletion reduced neuroinflammation and ASD-associated behaviors, supporting a gut–immune–brain axis (12). In the same study, microbial and metabolic regulators related to the glutamate/GABA ratio and 3-hydroxyglutaric acid were identified, and administration of *Limosilactobacillus reuteri* IMB015 reduced the glutamate/GABA ratio, neuroinflammation, and behavioral abnormalities (12).

This animal work is useful because it moves beyond associations and begins to connect gut microbiota with immune signaling, microglial response, neuronal activity, and behavior in a genetically susceptible model. It also supports the idea that genetic susceptibility and microbial environment may interact: the microbiome may not initiate ASD, but it may shape the severity or expression of ASD-related behaviors in vulnerable biological contexts. Thus, a gut–immune–brain model may be more informative than a simple gut–brain model. Immune cells, cytokines, and microglia may serve as key intermediates between microbial metabolites and neural outcomes.

However, animal models cannot fully capture the genetic, developmental, behavioral, dietary, and environmental heterogeneity of human ASD. Germ-free and BTBR mouse models are useful for testing possible mechanisms, but findings from these systems should be interpreted as mechanistic support rather than direct evidence that identical pathways operate in children with ASD. Translation to humans will require longitudinal studies and carefully designed interventions that include microbiome, metabolomic, immune, gastrointestinal, and behavioral outcomes.

## Microbiome-targeted interventions and precision approaches

Microbiome-targeted interventions should be discussed with caution. Current evidence does not support that probiotics or microbiota therapies treat ASD broadly. However, recent studies suggest that selected interventions may improve specific domains, especially gastrointestinal symptoms or social functioning in certain subgroups. Narula Khanna et al. conducted a single-blinded randomized controlled trial of 180 children with ASD and found that probiotic supplementation improved behavioral symptom severity and gastrointestinal symptoms compared with placebo (13). Mazzone et al. performed a pilot double-blind randomized placebo-controlled trial and found that *L. reuteri* improved social functioning but not overall autism severity or repetitive behaviors (14). Importantly, their translational findings also showed strain-specific effects in mice, with *L. reuteri* ATCC PTA 6475, but not DSM 17938, reversing social deficits (14).

A recent review by Takyi et al. summarized interventions targeting gut microbiota, including probiotics, prebiotics, synbiotics, antibiotics, antifungals, fecal microbiota transplant, microbiota transfer therapy, and diet (15). The review concluded that microbiota-targeted interventions are promising, particularly for gastrointestinal symptoms, but not yet established ASD therapies (15). Similarly, Wong et al., argued that microbiome biomarkers may eventually contribute to prediction, diagnosis, prognosis, and tailored therapies, while emphasizing that clinical causal evidence remains indirect and requires prospective validation in diverse cohorts (16). These findings indicate a precision-oriented approach: microbiome interventions may be most useful when matched to specific biological, gastrointestinal, behavioral, or metabolic profiles rather than applied to ASD as a single uniform condition.

Intervention studies also remain limited by small or moderate sample sizes, short follow-up periods, variation in probiotic strains and doses, inconsistent outcome measures, and differences in baseline gastrointestinal symptoms. Placebo effects and caregiver-reported outcomes may also influence behavioral findings. Importantly, benefits observed for gastrointestinal symptoms or social functioning should not be interpreted as evidence that probiotics treat ASD broadly. Future trials should define target subgroups before treatment, report diet and medication exposure, include standardized behavioral and gastrointestinal endpoints, and test whether microbiome or metabolite changes mediate clinical response. A summary of key human-centered and translational ASD microbiome studies is provided in Table 1.

**Table 1:** Summary of key human-centered and translational ASD microbiome studies.

Study	Sample/design	Main contribution	Key limitation
Su et al. (1)	Large pediatric cohort; 1,627 children	Identified multikingdom and functional gut microbiota markers in ASD.	Cross-sectional biomarker findings require independent validation.
Morton et al. (2)	Multi-dataset gut–brain axis analysis	Integrated microbiome, diet, metabolomic, cytokine, and brain-expression data.	Findings differed by matching strategy and dataset context.
Yang et al. (3)	Meta-analysis	Summarized recurring gut microbial differences between ASD and neurotypical children.	Substantial heterogeneity across cohorts and methods.
Manghi et al. (4)	Large oral microbiome analysis	Identified ASD-associated oral microbial and functional patterns.	Oral microbiome findings cannot be directly interpreted as gut findings.
Chen et al. (5)	Large pediatric multi-omics cohort	Linked microbial genomic variants and metabolites with altered host–microbe interactions.	Requires prospective validation and functional follow-up.
Liu et al. (6)	Pediatric stool cohort; 452 children	Suggested bacterial structural variants may improve diagnostic modeling.	Cross-sectional design limits causal inference.
Wu et al. (7)	Pediatric cohort; 818 children	Linked diet quality, food additives, GI symptoms, and ASD-related features with microbiome patterns.	Diet and behavior remain difficult to separate.
Lu et al. (8)	Multiplex ASD families	Highlighted strain-sharing and familial/environmental influences.	Family structure may limit generalizability.
Chen et al. (9)	Children with ASD and family members	Compared children with ASD, non-ASD siblings, and parents.	Shared household and genetic factors remain difficult to separate fully.
Wang et al. (10)	Preschool ASD/neurotypical cohorts	Linked GABA/glutamate imbalance and overrepresented *Escherichia* with mild ASD.	Mild ASD cohort; mechanistic translation remains limited.
Aziz-Zadeh et al. (11)	43 ASD and 41 neurotypical children	Connected tryptophan-related metabolites with brain activity and symptom measures.	Moderate sample size.
Narula Khanna et al. (13)	Single-blind randomized trial; 180 children	Reported probiotic-related improvement in GI and behavioral symptoms.	Single-blind design and need for replication.
Mazzone et al. (14)	Pilot double-blind randomized trial	Suggested strain-specific *L. reuteri* effects on social functioning.	Small pilot trial; not evidence of broad ASD treatment.
Takyi et al. (15)	Review of gut microbiota interventions	Summarized probiotics, prebiotics, synbiotics, antibiotics, fecal microbiota transplant, microbiota transfer therapy, and diet.	Intervention evidence remains heterogeneous and not yet established as broad ASD therapy.
Wong et al. (16)	Review of microbiome biomarkers	Discussed prediction, diagnosis, prognosis, and tailored therapy.	Clinical implementation requires prospective validation.

## Discussion and future directions

The current evidence allows for a cautiously optimistic view of the microbiome in ASD. Large human studies suggest that children with ASD often differ in microbial composition, microbial genes, microbial structural variants, diet–microbiome relationships, and metabolite profiles (1, 5–7, 10, 11). Meta-analytic evidence also supports differences in several gut microbial genera between ASD and neurotypical children, although findings remain inconsistent across studies (3). Large-scale oral microbiome work has also identified ASD-associated microbial markers and functional enrichment related to serotonin, GABA, and dopamine degradation pathways, although oral microbiome findings should not be interpreted as direct evidence of gut microbiome findings (4). Jointly, these studies indicate that microbiome alterations in children with ASD are repeatedly observed, but they are not simple, uniform, or independent of behavior, diet, and environment.

The next phase of ASD microbiome research should move toward a more precision-based approach. Efforts may prioritize longitudinal human cohorts beginning early in development, careful measurement of diet and medication exposure, gastrointestinal symptom tracking, metabolomics, immune profiling, sex differences, and standardized behavioral outcomes. Microbiome-targeted interventions need to be tested in biologically defined subgroups rather than broad ASD samples. For example, a child with severe constipation, food selectivity, altered tryptophan metabolism, and inflammatory markers may not respond the same way as a child without those features.

Sex differences also require more attention. ASD prevalence, immune signaling, neurodevelopmental trajectories, gastrointestinal symptoms, and microbiome composition may differ by sex, but many microbiome studies remain underpowered to test sex-specific effects. Future studies should report sex-stratified analyses when possible and consider whether microbiome-related pathways differ between males and females with ASD (12, 16).

Early-life and environmental factors also remain important for future research. Delivery mode, breastfeeding, antibiotic exposure, early-life infections, diet, medication exposure, and socioeconomic or geographic context may shape microbiome development and later neurodevelopmental outcomes. These factors should be measured carefully so that ASD-associated microbiome patterns can be separated from broader developmental and environmental influences (17).

Methodological standardization will be essential for improving reproducibility. Future ASD microbiome studies would benefit from standardized diagnostic characterization, ASD severity measures, gastrointestinal symptom assessment, dietary reporting, medication and antibiotic documentation, stool collection protocols, sequencing pipelines, metabolomic methods, and behavioral outcome measures. Greater standardization would make it easier to compare studies and determine whether observed microbiome patterns are reproducible across cohorts (18, 19).

Clinically, the translational value of ASD microbiome research may be strongest when microbiome measures are combined with gastrointestinal, metabolomic, immune, dietary, and behavioral data rather than used as stand-alone diagnostic tests. This approach is consistent with broader microbiome guidance emphasizing careful interpretation, standardization, and prospective validation before routine clinical implementation (19, 20).

**Figure 1:**
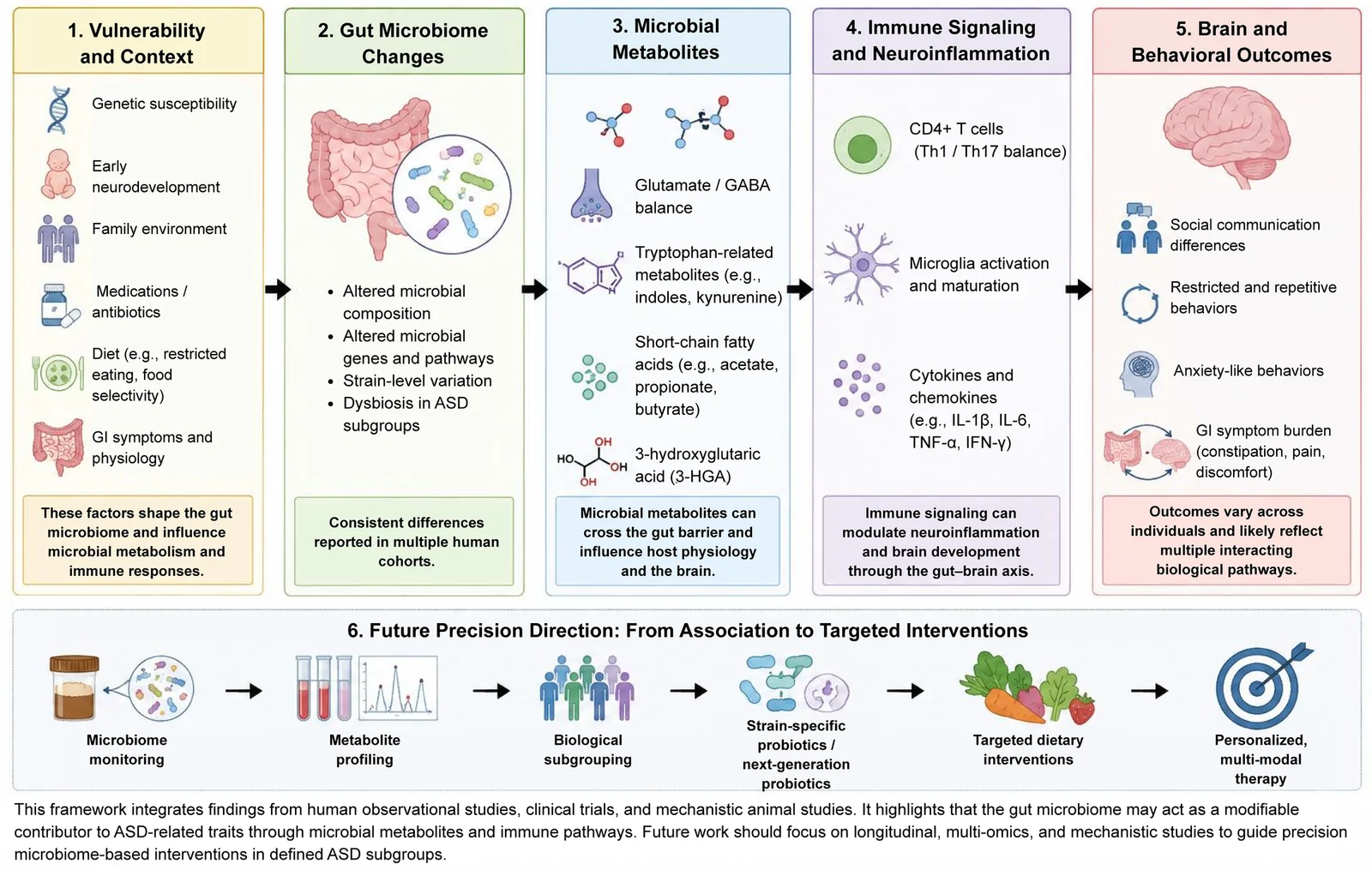
Proposed gut–immune–brain framework for ASD-related traits. This conceptual model integrates recent findings from human observational studies, clinical trials, and mechanistic animal studies. Genetic susceptibility, early neurodevelopment, family environment, medications, restricted eating, and gastrointestinal symptoms may shape gut microbiome composition and function. Microbial communities may influence ASD-related traits through metabolites such as glutamate/GABA-related pathways, tryptophan-related metabolites, short-chain fatty acids, and 3-hydroxyglutaric acid. These signals may affect immune pathways involving CD4+ T cells, microglia, cytokines, and neuroinflammation, ultimately influencing brain activity and behavioral domains. Rather than presenting the microbiome as a single cause of ASD, this framework may support future precision approaches using microbiome monitoring, metabolite profiling, biological subgrouping, strain-specific probiotics, and targeted dietary or microbial interventions. Created by the authors with Canva.

Overall, microbiomes should be viewed as promising and modifiable contributors to ASD-related biology, not as a single explanation for ASD. A gut–immune–brain framework offers a more scientifically balanced model: genetic and developmental vulnerability may interact with diet, microbial communities, microbial metabolites, immune signaling, and brain activity to influence specific ASD-related traits. This proposed framework is summarized in Figure 1. This framework may help move the field from association to targeted intervention, with the long-term goal of using microbiome monitoring, metabolite profiling, and strain-specific or diet-based therapies to support biologically defined subgroups of children with ASD.
